# Investigating barriers in HIV-testing oncology patients. The IBITOP study: phase I

**DOI:** 10.7448/IAS.17.4.19622

**Published:** 2014-11-02

**Authors:** Laurent Merz, Solange Peters, Stefan Zimmermann, Matthias Cavassini, Katharine Darling

**Affiliations:** 1 Service of Infectious Diseases, Lausanne University Hospital, Lausanne, Switzerland; 2 Service of Oncology, Lausanne University Hospital, Lausanne, Switzerland

## Abstract

**Background:**

Since the advent of combined antiretroviral therapy (ART), the incidence of non-AIDS-defining cancers (non-ADCs) among HIV-positive patients is rising. We previously described HIV testing rates of <5% in our oncology centre, against a local HIV prevalence of 0.4% [1]. We have since worked with the Service of Oncology to identify, how HIV testing can be optimized, we have conducted a study on investigating barriers in HIV-testing oncology patients (IBITOP) among treating oncologists and their patients.

**Methods:**

After an initial two-month pilot study to examine feasibility [2], we conducted the first phase of the IBITOP study between 1st July and 31st October 2013. Patients of unknown HIV status, newly diagnosed with solid-organ non-AIDS-defining cancer, and treated at Lausanne University Hospital were invited to participate. Patients were offered HIV testing as a part of their initial oncology work-up. Oncologist testing proposals and patient acceptance were the primary endpoints.

**Results:**

Of 235 patients with a new oncology diagnosis, 10 were excluded (7 with ADCs and 3 of known HIV-positive status). Mean age was 62 years; 48% were men and 71% were Swiss. Of 225 patients, 75 (33%) were offered HIV testing. Of these, 56 (75%) accepted, of whom 52 (93%) were tested. A further ten patients were tested (without documentation of being offered a test), which gave a total testing rate of 28% (62/225). Among the 19 patients who declined testing, reasons cited included self-perceived absence of HIV risk, previous testing and palliative care. Of the 140 patients not offered HIV testing and not tested, reasons were documented for 35 (25%), the most common being previous testing and follow-up elsewhere. None of the 62 patients HIV tested had a reactive test.

**Conclusions:**

In this study, one third of patients seen were offered testing and the HIV testing rate was fivefold higher than that of previously observed in this service. Most patients accepted testing when offered. As HIV-positive status impacts on the medical management of cancer patients, we recommend that HIV screening should be performed in settings, where HIV prevalence is >0.1%. Phase II of the IBITOP study is now underway to explore barriers to HIV screening among oncologists and patients following the updated national HIV testing guidelines which recommend testing in non-ADC patients undergoing chemotherapy.
